# Unravelling the antimicrobial action of antidepressants on gut commensal microbes

**DOI:** 10.1038/s41598-020-74934-9

**Published:** 2020-10-21

**Authors:** Yasmina Ait Chait, Walid Mottawea, Thomas A. Tompkins, Riadh Hammami

**Affiliations:** 1 NuGut Research Platform, School of Nutrition Sciences, Faculty of Health Sciences, University of Ottawa, Ottawa, ON K1N 6N5 Canada; 2 Department of Microbiology and Immunology, Faculty of Pharmacy, Mansoura University, Mansoura, Egypt; 3 Rosell Institute for Microbiome and Probiotics, Montreal, QC H4P 2R2 Canada

**Keywords:** Antimicrobials, Applied microbiology

## Abstract

Over the past decade, there has been increasing evidence highlighting the implication of the gut microbiota in a variety of brain disorders such as depression, anxiety, and schizophrenia. Studies have shown that depression affects the stability of gut microbiota, but the impact of antidepressant treatments on microbiota structure and metabolism remains underexplored. In this study, we investigated the in vitro antimicrobial activity of antidepressants from different therapeutic classes against representative strains of human gut microbiota. Six different antidepressants: phenelzine, venlafaxine, desipramine, bupropion, aripiprazole and (*S*)-citalopram have been tested for their antimicrobial activity against 12 commensal bacterial strains using agar well diffusion, microbroth dilution method, and colony counting. The data revealed an important antimicrobial activity (bacteriostatic or bactericidal) of different antidepressants against the tested strains, with desipramine and aripiprazole being the most inhibitory. Strains affiliating to most dominant phyla of human microbiota such as *Akkermansia muciniphila, Bifidobacterium animalis* and *Bacteroides fragilis* were significantly altered, with minimum inhibitory concentrations (MICs) ranged from 75 to 800 μg/mL. A significant reduction in bacterial viability was observed, reaching 5 logs cycle reductions with tested MICs ranged from 400 to 600 μg/mL. Our findings demonstrate that gut microbiota could be altered in response to antidepressant drugs.

## Introduction

The gut microbiota represents a diverse community relatively stable during the adult age^1^ that plays a crucial role in host physiology, homeostasis, development, and metabolism^2–4^. Over the past decade, there has been increasing evidence highlighting the implication of the gut microbiota in a variety of brain disorders such as depression, anxiety, and schizophrenia^5,6^. Studies have shown that depression affects the stability of gut microbiota, but the impact of antidepressant treatments on microbiota structure and metabolism remains underexplored^7^. Indeed, gut microbiota could be altered during major depressive episodes^7^ or in response to antidepressant treatments, which could be undervalued confounding factors^8–10^.

Antidepressant drugs have been increasingly shown to possess antimicrobial properties with possible implications in the microbiota-gut-brain axis. The anti-tuberculosis agent iproniazid was first to be used in the treatment of depression in the 1950s due to its euphoriant effects on tuberculosis patients, reviewed in^10^. Since then, several classes of antidepressants, including monoamine oxidase inhibitors (MAOIs), selective serotonin reuptake inhibitors (SSRIs), *N*-methyl-d-aspartate (NMDA) receptor antagonists, and tricyclic antidepressants (TCAs) have been assessed for their antimicrobial potency, with their mechanism of action being poorly investigated. For instance, SSRIs such as sertraline, fluoxetine and paroxetine are efflux inhibitors in bacteria cell walls and are effective on Gram-positive bacteria such as *Enterococcus* and *Staphylococcus*^11^. In addition, several studies highlighted the antifungal potential of SSRIs fluoxetine, sertraline, and paroxetine against *Aspergillus* spp., *Candida parapsilosis*, and *Candida albicans*^11–13^. In addition, several SSRIs have been reported to have antimicrobial properties at high concentrations while having antimicrobial enhancer properties at lower concentrations. This synergistic effect is confirmed by decreases in the minimum inhibitory concentrations of antibiotics when combined with antidepressants^14^. Likewise, Ketamine, an NMDA antagonist, was shown effective against *Staphylococcus aureus*, *S. epidermidis*, *Enterococcus faecalis*, *Streptococcus pyogenes*, and *Pseudomonas aeruginosa*, and *Candida albicans*^15^. Another class of antidepressant drugs, the TCAs, was reported to have anti-plasmid effects and to prevent the growth of intestinal pathogens such as *E. coli*, *Yersinia enterocolitica*, *Giardia lamblia*, *Plasmodium falciparum*, and *Leishmania* spp, reviewed in^10^.

Besides, other evidence gathered from animal studies suggested that the antidepressants modulate the composition of the intestinal microbiota^9,16–18^. Administration of TCA desipramine causes important side effects and results in a higher incidence of infections generating gingivitis and dysbiosis of oral microbiota^19^. A prior study revealed that ketamine also modulates the fecal microbiome in the susceptible mice after chronic social defeat stress, suggesting an antidepressant mechanism partly mediated by the modulation of gut microbiota^20^. However, all the existing previous studies were carried out in animal models or using isolated strains (references or clinical isolates) that do not necessarily represent the human gut microbiota to enhance the efficacy of existing chemotherapeutic agents such as antibiotics. Limited studies have investigated the effect of antidepressant medications on the growth of commensal microbial residents of the human gut microbiota. For instance, of 1000 non-antibiotics drugs, oral antipsychotics were able to reduce the in vitro growth of gut bacterial strains^21^. Likewise, Cusotto et al.^9^ reported the in vitro sensitivity of two commensal bacteria, notably *Escherichia coli* APC105 and *Lactobacillus rhamnosus* 6118 toward two SSRIs, fluoxetine and escitalopram.

The chronic use of antidepressant drugs presenting antimicrobial effects may be related to the development of adaptive alterations in gut microbiota, with potentially deleterious effects^10^. The purpose of the present study was to investigate the antimicrobial effect of some oral commonly prescribed antidepressants from different therapeutic classes against commensal bacteria representative of the predominant phyla found in the human gut microbiota.

## Results

### Antibacterial activity on solid media

**Figure 1:**
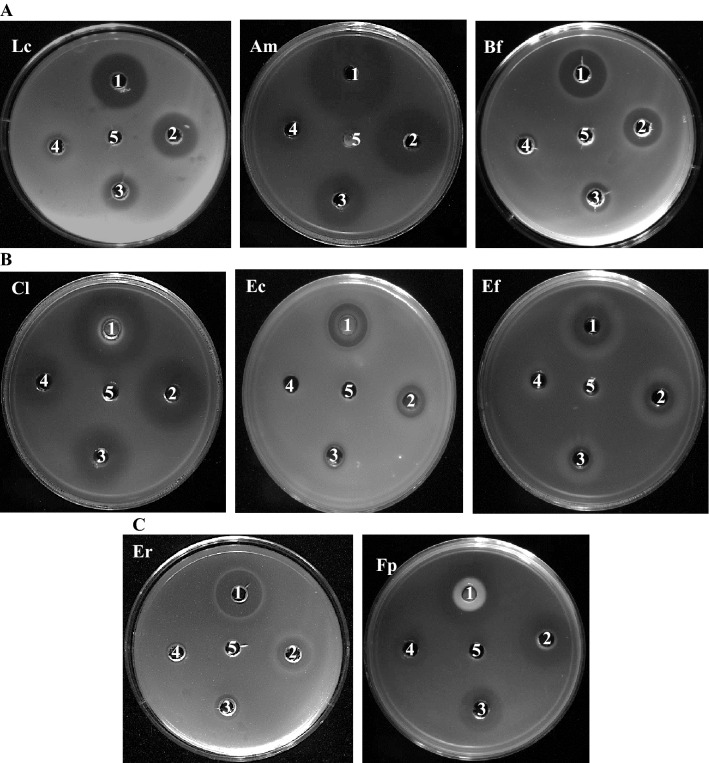
Inhibition zone of desipramine (**A**), aripiprazole (**B**) and phenelzine (**C**) against some intestinal bacteria strains. Lc: *L. casei*, Am: *A. muciniphila*, Bf: *B. fragilis*, Cl: *C. leptum*, Ec: *E. coli*, Ef: *E. faecium*, Er: *E. rectale*, Fp: *F. prausnitzii*. (1: 10 mg/mL; 2: 5 mg/mL; 3: 2.5 mg/mL; 4: 1.25 mg/mL; 5: 0.625 mg/mL).

The antibacterial activity of different doses (0.625–10 mg/mL) of the tested antidepressants was first assessed by the well diffusion method. As shown in Fig. 1, these drugs display a dose- and drug-dependent antibacterial effect. Desipramine and aripiprazole showed the most inhibitory effect against all the tested strains with respective diameter of inhibition zone ranging from 13 to 35 mm and 15 to 31 mm (Table S2). At lesser extent, phenelzine and (s)-citalopram showed moderate antibacterial activity against some intestinal strains with diameter inhibition zone going from 9 to 19 mm (Table S3). Besides, minimal inhibition zone (around 9 mm) was observed in some strains with bupropion; however, no zone was detected with venlafaxine (data not shown). *Akkermansia muciniphila* and *Clostridium leptum* were the most sensitive strains to tested antidepressants (Fig. 1), while *Lactobacillus rhamnosus* being the most resistant (Table S3).

### Growth kinetics and determination of minimum inhibitory concentrations (MICs)

**Figure 2:**
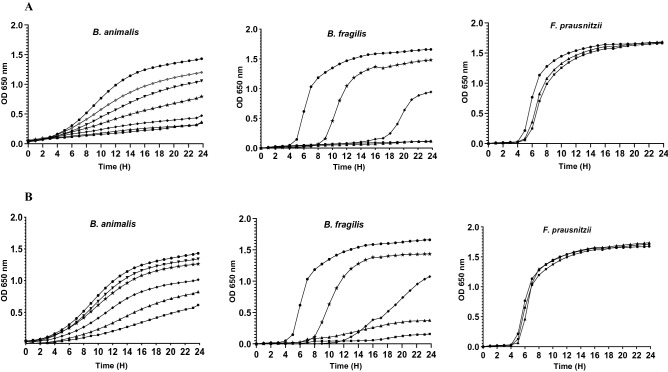
Growth of some intestinal strains in the presence of (**A**) Desipramine and (**B**) Aripiprazole. Concentrations (μg/mL) of antidepressants were 0 (circle), 800 (square), 600 (triangle), 400 (diamond), 300 (star), 200 (inverted triangle) and 150 (cross).

**Figure 3:**
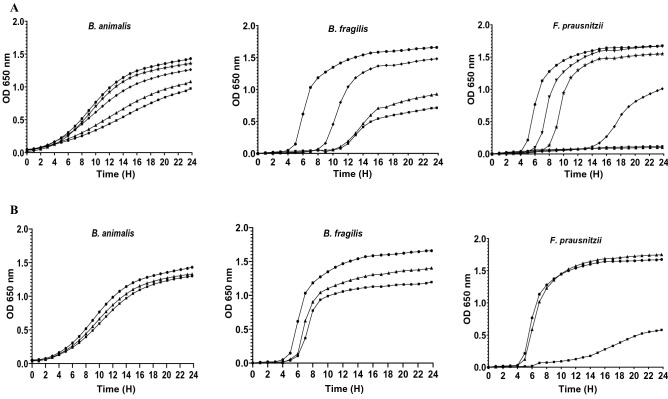
Growth of some intestinal strains in the presence of (**A**) Phenelzine and (**B**) (*S*)-citalopram. Concentrations (μg/mL) of antidepressants were 0 (circle), 800 (square), 600 (triangle), 400 (diamond), 300 (star), 200 (inverted triangle) and 150 (cross).

**Figure 4:**
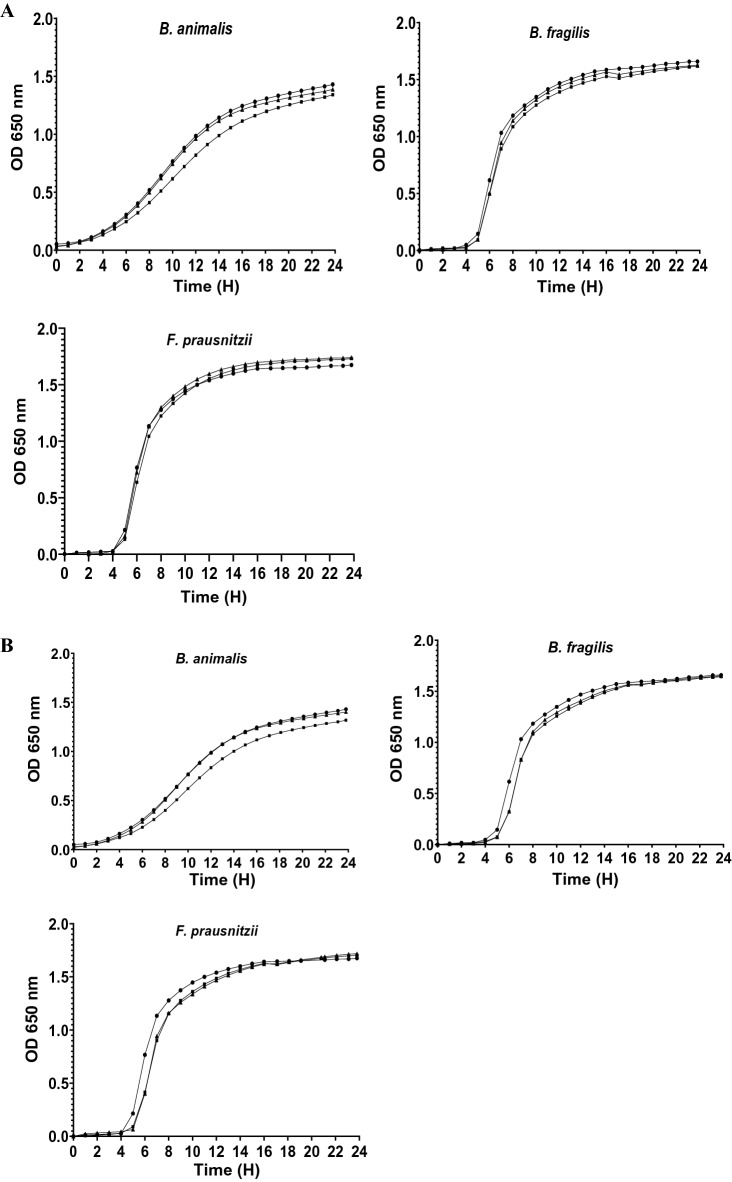
Growth of some intestinal strains in the presence of (**A**) Venlafaxine and (**B**) Bupropion. Concentrations (μg/mL) of antidepressants were 0 (circle), 800 (square), 600 (triangle), 400 (diamond), 300 (star), 200 (inverted triangle) and 150 (cross).

The antimicrobial activity of tested antidepressants against 12 commensal intestinal strains was quantified using the micro-broth dilution method. Figures 2, 3 and 4 illustrate the growth kinetics of some commensal gut bacteria. In the presence of increasing concentrations of antidepressants, the growth curves were dose-dependent, with strains being totally or partially inhibited. Desipramine was very active against most of the tested intestinal strains (10/12) with MIC values varying from 75 to 800 μg/mL (Table 1). For instance, 75 μg/mL of desipramine was enough to inhibit the growth of *A. muciniphila* partially, while higher concentrations (> 150 μg/mL) inhibited its growth completely. *Faecalibacterium prausnitzii* and *Eubacterium rectale* were the least susceptible to desipramine (MIC > 800 μg/mL). The tested strains were also sensitive to aripiprazole at MIC values ranging from 200 to 800 μg/mL. Beside *A. muciniphila* which was highly sensitive to aripiprazole (MIC = 200 μg/mL), other bacteria including, *Lactobacillus casei, Enterococcus faecium, Bacteroides fragilis*, and *C. leptum* were all inhibited at a dose of 300 μg/mL (Table 1). Comparatively, phenelzine exhibited a higher inhibitory effect against *E. rectale* and *F. prausnitzii* with respective MIC values of 300 and 400 μg/mL, while (s)-citalopram inhibited *E. rectale* at MIC value of 300 μg/mL. At the highest tested concentration (800 μg/mL), venlafaxine and bupropion presented MICs > 800 μg/mL or no inhibitory activity against the tested strains.

**Table 1:** Minimal inhibitory concentration (MICs) of antidepressants against commensal gut bacteria.

Intestinal strains	Minimal inhibitory concentration (μg/mL)					
	Phenelzine	Venlafaxine	Desipramine	Bupropion	Aripiprazole	Citalopram
*L. reuteri* ATCC 23272	> 800	NI	600	NI	800	> 800
*L. rhamnosus* ATCC 53103	> 800	NI	800	NI	600	> 800
*L. casei* ATCC 393	> 800	NI	300	> 800	300	800
*B. animalis* ATCC 25527	800	> 800	200	> 800	400	> 800
*E. faecium* ATCC 35667	> 800	800	600	> 800	300	> 800
*E. rectale* ATCC 33656	300	NI	> 800	> 800	> 800	300
*F. prausnitzii* ATCC 27768	400	NI	> 800	> 800	> 800	800
*B. fragilis* ATCC 25285	600	> 800	400	> 800	300	800
*P. aeruginosa* ATCC 27853	600	800	300	800	600	800
*E. coli* ATCC 25922	800	NI	150	> 800	600	800
*C. leptum* ATCC 29065	800	> 800	300	> 800	300	800
*A. muciniphila* ATCC BAA-835	> 800	> 800	75	> 800	200	> 800

*NI* no inhibition at the maximal tested concentration (800 μg/mL).

### Bacteria viability in the presence of different antidepressant concentrations

**Figure 5:**
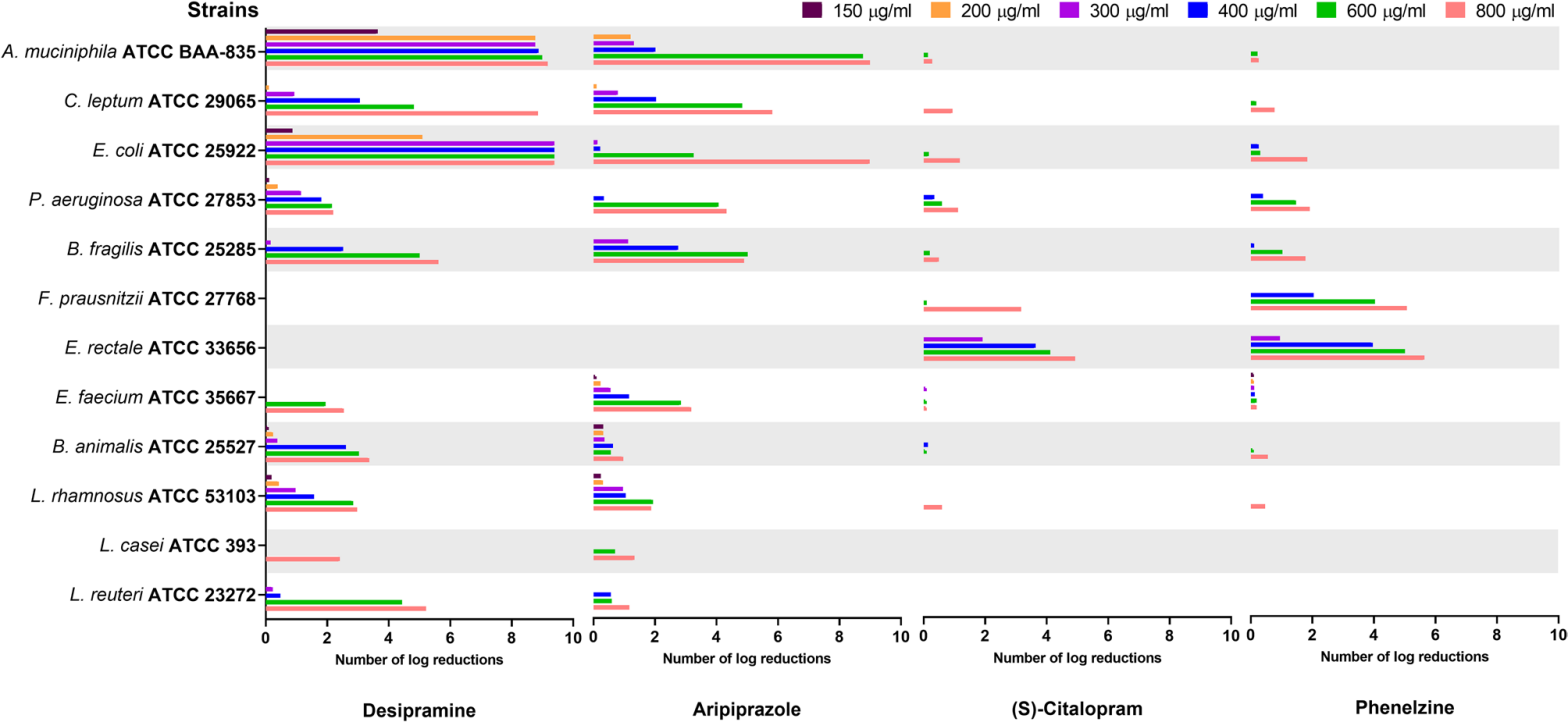
Logarithmic reductions of the growth of the reference strains in the presence of the antidepressants.

The logarithmic reductions of the viable bacteria (colony forming units: CFU) in presence of antidepressants after 16 h incubation is presented in Fig. 5. Increased doses of antidepressants significantly reduced CFU counts (*P* < 0.05). For instance, desipramine strongly and significantly (*P* < 0.05) affected the viability of tested intestinal strains, being strain-dependant. Indeed, colonies of *A. muciniphila* and *E. coli* was completely inhibited at respective concentrations of ≥ 300 and 200 μg/mL of this drug (Fig. 5). No viable *A. muciniphila* colonies were detected in samples treated with either desipramine or aripiprazole at 600 or 800 μg/mL compared to initial inoculum counts (5.58 ± 0.19 log_10_ cfu/mL), suggesting a bactericidal effect of these drugs (Figure S2C). At concentrations up to 300 μg/mL of desipramine, the CFU decreased significantly (*P* < 0.05) by more than 5 log cycles (i.e. *L. reuteri* and *B. fragilis* at 800 μg/mL), 4 log cycles (i.e. *C. leptum* at 800 μg/mL and *L. reuteri* at 600 μg/mL), 3 log cycles (i.e. *B. animalis* at 600 μg/mL), 2 log cycles (i.e. *P. aeruginosa* at 600 μg/mL), 1 log cycle (i.e. *L. casei* at 400 μg/mL) or less than 1 log (i.e. *B. animalis* at 300 μg/mL). No reduction was detected against the two strains *E. rectale* and *F. prausnitzii.* Likewise, aripiprazole completely inhibited the colonies of *E. coli* at 800 μg/mL and *A. muciniphila* at 800 and 600 μg/mL (Fig. 5; Figure S2C). Reductions of 5 log cycles were obtained for some of the other intestinal strains. The number of *E. rectale* and *F. prausnitzii* was not affected by the different concentrations of aripiprazole. Comparatively, phenelzine and (s)-citalopram (Fig. 5) were more active towards *E. rectale* and *F. prausnitzii*, with reductions varying from 2 to 5 logs when tested at 400 μg/mL or up. No significant reductions were obtained in the presence of different concentrations of venlafaxine and bupropion (Figure S1).

## Discussions

There is an increasing interest on how therapeutic drugs could affect and alter the human gut microbiota composition and function^21^. While some knowledge is accumulating on the antimicrobial impact of some antidepressants on isolated strains or the gut microbiota of animal models, information about other classes of antidepressants and representative species from the human gut is poorly investigated. There is an urgent need to clarify the real contribution of the antimicrobial role of antidepressants and the subsequent consequences to gut microbiota structure and metabolism. In this study, we investigated the in vitro effect of commonly prescribed antidepressants from different classes on commensal bacterial strains members of the human gut microbiota. The results clearly demonstrated that most of the tested antidepressants exerted an important dose-dependent inhibitory effect (bactericidal in some cases) on the growth of the tested bacterial strains.

Desipramine, belonging to tricyclic antidepressants class, showed the most potent antibacterial activity and significant (*P* < 0.05) growth reduction, with *A. muciniphila* (Verrucomicrobia family) and *E. coli* (Proteobacteria group) being the most sensitive microorganisms at MIC values of 75 and 150 μg/mL, respectively. Information about the antimicrobial activity of desipramine, in particular toward human gut strains, is missing from the literature. In an in vivo study^17^, reported that desipramine was able to reduce the richness and increase beta diversity of Male BALB/c mice gut microbiota. Also, the same authors found a reduction in abundance at the genus level of *Ruminococcus*, *Adlercreutzia*, and an unclassified Alphaproteobacteria in mice treated with desipramine. Likewise, administration of desipramine was also shown to cause important side effects and results in a higher incidence of infections generating gingivitis and dysbiosis of oral microbiota^19^. Other representatives of the tricyclic antidepressants group were previously shown to possess an in vitro antimicrobial effect toward human pathogenic species, such as amitriptyline against *Staphylococcus* spp., *Bacillus* spp., and *Vibrio cholerae*^22^; and imipramine, which inhibited the growth of *E. coli* and *Yersinia enterocolitica*^23^. Another FDA-approved TCA drug, maprotiline, has shown the potential to reduce the severity of *Francisella* infection by decreasing virulence without being bactericidal^24^. Maprotiline and chlorpromazine have strong antibiofilm activity against *Francisella*^24^. Besides its antibiofilm inhibitory activity in *Salmonella* Typhimurium and *Francisella novicida*, chlorpromazine is strongly inhibitory to *F. novicida* growth^24^. Moreover, TCA amoxapine was demonstrated to resensitize methicillin-resistant *S. aureus* to oxacillin in vitro^25^. In addition, members of TCA drugs were reported to possess anti-plasmid effects and to inhibit intestinal pathogens such as *E. coli*, *Yersinia enterocolitica*, *Giardia lamblia*, *Plasmodium falciparum*, and *Leishmania* spp, reviewed in^10^.

Interestingly, aripiprazole and bupropion, belonging both to the atypical group of antidepressants, displayed different effects, with aripiprazole having a pronounced antibacterial activity and bupropion exhibiting no significant growth inhibition. Some previous studies have shown that aripiprazole exerts an inhibitory effect on gut microbiota^9,21^. For instance, Maier et al.^21^ demonstrated, in an in vitro large-scale study, that several non-antibiotics drugs inhibit the growth of human gut bacteria, with *Akkermansia* levels being reduced in the presence of atypical antipsychotics (including aripiprazole). Our study revealed a high antibacterial sensitivity of *Akkermansia* to aripiprazole and desipramine, with bactericidal effects at tested concentration range. In rats, the administration of aripiprazole for 4 weeks was associated with modulation of the relative abundance of firmicutes genera, including *Clostridium*, *Ruminiclostridium*, *Intestinibacter* and *Eubacterium coprostanoligens*^9^.

Being the most commonly prescribed class of antidepressants to treat the major depressive disorder, the selective serotonin reuptake inhibitors (SSRIs) tested in this study, (*S*)-citalopram was found to be more active against *E. rectale* and *F. prausnitzii,* both belonging to the Firmicutes phylum. Many isolated in vitro studies conducted with SSRI drugs, using reference strains or clinical isolates (not necessarily representing the gut microbes), showed an antimicrobial effect^10^. According to^9^, Escitalopram, an enantiomer of (*S*)-citalopram, and fluoxetine were able to completely inhibit the growth of *E. coli* APC105 and *L. rhamnosus* 6118, both resident of the human gut, at a concentration of 600 μg/mL. In addition, citalopram was reported to exert an inhibitory effect against some pathogenic strains of *E. coli* with MIC value over than 800 μM^26,27^, and against *P. aeruginosa* strains with MIC ranged 4000–6000 mg/mL^27^. Other studies were demonstrated the antimicrobial effect of more SSRIs drugs such as sertraline, fluoxetine, citalopram and paroxetine on *Staphylococcus*, *Enterococcus, Pseudomonas, Bacillus* and *Clostridium* strains^11,28,29^. In addition, several studies highlighted the antifungal potential of SSRIs fluoxetine, sertraline, and paroxetine against *Aspergillus* spp., *Candida parapsilosis*, and *Candida albicans*^12,13,27^. Several SSRIs have been reported to have antimicrobial properties in high concentrations while having antimicrobial enhancer properties in lower concentrations. This synergistic effect is confirmed by decreases in the minimum inhibitory concentrations of antibiotics when combined with antidepressants. For example, sertraline has been shown to affect bacterial transcription and increase the susceptibility of resistant *Escherichia coli* APEC_O2 to tetracycline in vitro^14^. Nevertheless, high-dose treatments with sertraline as an adjuvant for the treatment of antibiotic-resistant *E. coli* infections were reported to exacerbate the pathological outcome of infection in chickens^30^. Other studies using animal models provided in vivo evidence for the antimicrobial activity of SSRIs^9,17,31^.

Venlafaxine from the therapeutic class of serotonin-norepinephrine reuptake inhibitors (SNRIs) did not show any antibacterial effect on the growth of tested bacterial strains. This finding is in agreement with^9^ who tested in vitro against *L. rhamnosus* and *E. coli* at maximal concentrations of 600 μg/mL. Moreover, venlafaxine was found to be inactive when tested against *E. coli* and *P.* a*eruginosa*; however, this drug augmented the antibacterial effects of antibiotics towards resistant strains^27^.

The monoamine-oxidase inhibitor (MAOIs), Phenelzine**,** showed a remarkable antibacterial effect on some strain’s representative of the firmicutes phylum (*E. rectale* and *F. prausnitzii*). Little is known about the antimicrobial activity of phenelzine. This may be explained by the more consideration directed to other classes of antidepressants.

Variation in the antibacterial activity of antidepressants between the different therapeutic classes was observed in this study, suggesting potential differences in their mechanisms of inhibitory action. Even these latter are not fully understood, the one proposed mechanism for the action of SSRIs is inhibition of efflux pumps^28^ and decrease of the activity of DNA gyrase for TCAs antidepressants^10^. The mechanisms underlining the drug-induced alterations in gut microbiota are only partly known. Indeed, the findings from this work highlight the variability in MIC values of antidepressants towards the strains of different species where some antidepressants found to exhibit a bacteriostatic or bactericidal effect. These differences in microbe’s inhibition may facilitate the intestinal abundance changes by selecting some bacteria and promote the overgrowth of others, causing a shift of microbial communities towards dysbiosis or eubiosis.

The antimicrobial activity of antidepressant against gut microbiota could be considered as a side effect, but also possibly as mechanism of antidepressant action in the gut. Indeed, while lanicemine does not exhibit antidepressant effects in treatment-resistant depressed patients, ketamine shows rapid and sustained antidepressant effects, both being NMDAR antagonists^20^. Ketamine modulates the fecal microbiome in the susceptible mice after chronic social defeat stress, suggesting an antidepressant mechanism partly mediated by the modulation of gut microbiota^20^. Therefore, the antimicrobial effect of antidepressants could be also an important mechanism for alleviating intestinal dysbiosis observed in patients with MDD^10^.

Importantly, we should also take into consideration that the concentrations below the MICs or the sub-inhibitory MICs, even they did not inhibit the growth of the intestinal strains, they delayed their growth in the first hour of incubation triggering the reduction of the growth rate. It was proved in case of antibiotics that the continuous growth in the presence of sub-inhibitory concentrations could select resistant bacteria and promote the evolution of resistance development^32^. Moreover, the antidepressants impaired differentially specific microbiota genera that are commonly correlated with human health and dysbiosis. For example, *B. fragilis* member of Bacteroidetes family has been shown to have beneficial roles such as stimulating immune development^33^. Bifidobacteria are known for their ability to protect the gut, boost the immune system, and control inflammatory responses^34^.

An important aspect of the current study is to extrapolate the in vitro findings to the human gut level in a way to understand the link between antipsychotic-induced microbiota dysbiosis and metabolic dysfunction. In fact, very few observational studies in humans have examined the behaviour of the gut microbiome following antidepressant treatment. The chronic use of the atypical antipsychotic, risperidone, in children, gradually decreased the Bacteroidetes: Firmicutes ratio, which is associated with a mass body gain^35^. Additionally, Flowers et al.^36^ demonstrated that, in adult subjects with bipolar disorder, the atypical antipsychotic (AAP) class increased significantly *Lachnospiraceae* family abundance and decreased *Akkermansia* genus. This latter species is known to have beneficial anti-inflammatory properties and can protect against gut barrier dysfunction and fat mass development^37^. More recently, an increase in fecal microbiota biodiversity, mainly alpha diversity was shown in human patients after six weeks of concomitant therapy using 5–20 mg of escitalopram^38^.

To convert the in vitro observations to in vivo human gut level, we need to understand whether the antipsychotics medications reach the gastrointestinal tract (GIT) at sufficient concentrations to exert an antimicrobial effect. It is difficult to estimate the real concentrations of orally administered psychotropics in the human GIT since these drugs are affected by many factors like dose, solubility, distribution of fluids volume, transit time and uptake and metabolization by human cells and by bacteria. In this work, we focused on the effective concentrations in the colon (specifically ascendant colon), a part of the GIT that grows the most abundant microbial populations and essential site of fermentation^39^, and the colon concentrations were estimated based on fecal excretion data gathered from previously published works and DrugBank^40^, and the maximal daily doses for each antidepressant (please refer to Table S4). To calculate the approximative colonic concentrations, we assumed the volume of the colon from two different studies. Schiller et al.^41^ reported the mean fluid colon volume after a meal being 18 mL, while Pritchard et al.^42^ reported a volume of 480 mL. As we can observe from Table S4and according to^41^, the estimated concentrations are higher than the tested concentrations in this study. For example, if we assumed the minimum daily concentration for desipramine as 25 μg/mL, and the remaining amount in feces as 30%, the approximative concentration in the colon will be 1389 μg/mL, 16-fold higher than the highest concentration tested for desipramine in this work. Despite the difference in colonic volume reported in reference studies^41,42^, the real drug concentration depends on how much of free-water available to dissolve the chemicals and should be somewhere in between, thus requiring more research. Another point to take into consideration is that the proportions excreted in feces given for the different antidepressants do not reflect the real remaining amount of these drugs, because of the gradual solubilization through the passage from the different sections of the colon. This means that higher exposure will be in the ascendant colon, which represent the microbial-enriched regions^39^. Also, the exposure time and the cumulative effect of drugs in the colon may be a determinant factor to increase the risk of antimicrobial activity, since the antidepressants are taken daily and for an extended period. Indeed, according to Pratt et al.^43^, 25% of individuals in the USA have used antidepressants for more than 10 years between 2011 and 2014. In addition, considering the clinical context, where polypharmacy and comorbidities play an important role, combination of several drugs even at low concentration could influence the gut microbiota structure and function^44^. Beside antibiotics, non-antibiotic drugs can also contribute to antimicrobial resistance^21,44^. This impact on resistome profile seems likely considering the long-term use of antidepressants.

Little is known about the microbial drug metabolising enzymes in the GIT that could influence both compound structure and microbiome profile. Gut microbiota in general can metabolize xenobiotics either directly, mainly through reduction or hydrolysis, or indirectly through affecting host drug metabolism^45^. Some of the bacterial strains employed in our study are known to metabolize other drugs. For example, *Lactobacillus*, *Bacteroides*, and *Enterococcus* spp. were capable of metabolizing sulfasalazine via reduction^46^). However, no evidence is available regarding their effect on the tested antidepressants degradation, but we can assume that the bacterial-xenobiotic crosstalk is bidirectional and can apply to our case of psychotropics.

Finally, multiple studies have reported albeit confusing changes in microbiome abundance in depression. For instance, Mason et al.^47^ have recently reported a depletion of *C. leptum* and *Bacteroides* in depression and anxiety, but no information is reported about medication. Of note, *A. muciniphila*, *C. leptum*, and *B. fragilis*, important anti-inflammatory microbial groups, were found in our study very affected by tested antidepressants. Therefore, the impact of antidepressant drugs should be included in the equation as confounding factors when investigating microbial biomarkers, knowing that patients with MDD are usually put in long-term antidepressant medication.

## Conclusion

Our findings indicate clearly the strong antimicrobial effect of antidepressants from different chemical classes against gut commensal bacteria representative of the predominant phyla found in the human gut microbiota. The chronic use of antidepressant drugs presenting antimicrobial effects may be related to the development of adaptive alterations in gut microbiota, with potential deleterious effects. The antimicrobial activity of antidepressant against gut microbiota could be considered as a side effect, but also possibly as mechanism of antidepressant action in the gut. There is an urgent need to clarify the impact and mechanisms of the antimicrobial activity of antidepressants and the subsequent consequences to gut microbiota structure and metabolism. The present study provides new insights into the existing interplay between psychotropic chemicals and microbiota while further investigations are still needed.

## Material and methods

### Antidepressants

The six (6) antidepressants tested in this study are listed in Table 2. The choice of these drugs was based on their common prescription, mode of action, and therapeutic class. Venlafaxine hydrochloride, bupropion hydrochloride, aripiprazole and (*S*)-citalopram oxalate were purchased from TCI America (Portland, USA) while phenelzine sulphate salt and desipramine hydrochloride were from Sigma Aldrich (St. louis, MO, USA). The stock solutions were prepared following the manufacturer’s recommendations (please refer to Table S2) to achieve a concentration of 10 mg/mL, filter sterilized and then stored at − 20 °C until use. Working solutions of 1.8 mg/mL and 1.2 mg/mL were prepared from the stock solutions in the respective growth media before each experiment.

**Table 2:** List of antidepressants tested in this study.

Drug	Class	Mode of action
Phenelzine	Monoamine-oxidase inhibitor (MAOIs)	Inhibition of the breakdown of neurotransmitters (norepinephrine, serotonin, dopamine) by blocking the monoamine oxidase enzyme
Venlafaxine	Serotonin-norepinephrine reuptake inhibitors (SNRI)	↑ synaptic levels of 5-HT and NE by blocking the reuptake of the neurotransmitters into the presynaptic neuron
(S)-Citalopram	Serotonin-specific reuptake inhibitors (SSRI)	↑ synaptic levels of 5-HT by blocking the reuptake of the neurotransmitter into the presynaptic neuron
Desipramine	Tricyclic antidepressants (TCA)	Inhibition of noradrenaline and serotonin reuptake by neurons
Bupropion	Atypical antidepressants	It has predominantly antagonist activity on postsynaptic D2 (Dopamine) receptors and partial agonist activity on presynaptic D2 receptors
Aripiprazole	Atypical antidepressants	It has predominantly antagonist activity on postsynaptic D2 (Dopamine) receptors and partial agonist activity on presynaptic D2 receptors

Upward arrow signifies increase, 5-HT serotonin, *D* dopamine.

### Media, bacterial strains and culture conditions

De Man, Rogosa and Sharpe (MRS), Brain Heart Infusion (BHI) and Fastidious Anaerobe Broth (FAB) media were obtained from Criterion (Santa Maria, CA, USA). Mucin from porcine stomach and yeast extract were purchased from Sigma (St. Louis, MO, USA). Twelve (12) commensal bacterial strains were purchased from the American Type Culture Collection (Table S1). The intestinal strains were selected in a way to represent the main abundant phyla in the human gut, notably Bacteroidetes, Firmicutes, Actinobacteria, Proteobacteria and Verrucomicrobia. All strains were cultured in their recommended media (Table S1). Frozen stocks of strains were maintained at − 80 °C until use. Bacteria strains were grown in their appropriate media a least three times at 37 °C before each experiment to obtain a robustly and uniformly growing culture. The enzyme Oxyrase for broth purchased from Sigma Aldrich (St. Louis, MO, USA) was added to culture media at 1% to promote the growth of the anaerobic strains.

### Determination of the antibacterial activity

#### Agar well diffusion method

The antimicrobial activity of antidepressants was determined visually using the agar well diffusion assay, as previously described^48^. Briefly, appropriate media containing 7.5 g agar/L was cooled to 45 °C, seeded with an overnight culture of each intestinal strain at 1% (v/v) and poured into a sterile Petri dish (25 mL). After solidification, 7 mm diameter wells were made using the wide end of a sterile glass pipette and filled with 80 μL of ½ dilution series of antidepressant solutions starting from 10 mg/mL to 0.625 mg/mL. The plates were kept at 4 °C for 2 h and then incubated for 24 h at 37 °C. The diameter of the inhibition zone around the well was measured.

#### Broth microdilution method and determination of Minimum Inhibitory Concentration

The antibacterial effect of the antidepressants was performed using the broth microdilution method as described in the approved CLSI standard reference method for antimicrobial susceptibility testing by broth diffusion for aerobic^49^ and anaerobic bacteria^50^. Briefly, a 96-well microplate (Randor, PA, USA) was filled by distributing 100 μL of appropriate media (corresponding to each strain). Medium alone and medium with each strain inoculum were used as a negative and positive control on the same microplate. Then, 100 μL of each working antidepressant solution was added to each well (C1 to C12) and twofold serially diluted to reach final concentrations ranged from 800 to 150 μg/mL. Wells thus containing 100 μL of media were inoculated with 100 μL overnight culture of the intestinal strains diluted to a concentration of 10^5^–10^6^ CFU/mL. Plates were incubated anaerobically at 37 °C for 24 h. Reading of each well was performed with measuring the optical density (OD) at 650 nm in a microplate reader (Tecan Spark, Austria)^48^. The MIC of each antidepressant was determined as the lowest concentration that inhibits the growth of the target strain. Also, samples incubated for 16 h were centrifuged to remove the drug-containing media and resuspended in the appropriate drug-free media. The viable bacterial strains were then determined by standard plate counting method on the appropriate media solidified with agar 1.2% and expressed as colony-forming units (CFU) per ml.5.4. Statistical analysis.

Statistical analysis was performed using GraphPad Prism v8.3. Data were expressed as the mean ± standard deviation (SD) of triplicate experiments. One-way analysis of variance (ANOVA) followed with Tukey's multiple comparison was applied to determine the statistically significant difference (*P* < 0.05) among experimental variables.

## Supplementary information

Supplementary Information.

## References

[1] Mehta RS (2018). Stability of the human faecal microbiome in a cohort of adult men. Nat. Microbiol..

[2] Bauer KC, Huus KE, Finlay BB (2016). Microbes and the mind: emerging hallmarks of the gut microbiota–brain axis. Cell. Microbiol..

[3] Sampson TR, Mazmanian SK (2015). Control of brain development, function, and behavior by the microbiome. Cell Host Microbe.

[4] Turnbaugh PJ (2009). A core gut microbiome in obese and lean twins. Nature.

[5] Codagnone MG (2019). Programming bugs: microbiota and the developmental origins of brain health and disease. Biol. Psychiatry.

[6] Kelly JR (2016). Transferring the blues: depression-associated gut microbiota induces neurobehavioural changes in the rat. J. Psychiatr. Res..

[7] Jiang H (2015). Altered fecal microbiota composition in patients with major depressive disorder. Brain. Behav. Immun..

[8] Cheung SG (2019). Systematic review of gut microbiota and major depression. Front. Psychiatry.

[9] Cussotto S (2019). Differential effects of psychotropic drugs on microbiome composition and gastrointestinal function. Psychopharmacology.

[10] Macedo D (2017). Antidepressants, antimicrobials or both? Gut microbiota dysbiosis in depression and possible implications of the antimicrobial effects of antidepressant drugs for antidepressant effectiveness. J. Affect. Disord..

[11] Ayaz M (2015). Sertraline enhances the activity of antimicrobial agents against pathogens of clinical relevance. J. Biol. Res.-Thessalon..

[12] Gu W, Guo D, Zhang L, Xu D, Sun S (2016). The synergistic effect of azoles and fluoxetine against resistant *Candida albicans* strains is attributed to attenuating fungal virulence. Antimicrob. Agents Chemother..

[13] Costa Silva RA (2017). In vitro anti-Candida activity of selective serotonin reuptake inhibitors against fluconazole-resistant strains and their activity against biofilm-forming isolates. Microb. Pathog..

[14] Li L, Kromann S, Olsen JE, Svenningsen SW, Olsen RH (2017). Insight into synergetic mechanisms of tetracycline and the selective serotonin reuptake inhibitor, sertraline, in a tetracycline-resistant strain of *Escherichia coli*. J. Antibiot. (Tokyo).

[15] Evrensel A, Ceylan ME, Kim Y-K (2018). Gut-Microbiota-Brain Axis and Depression. Understanding Depression.

[16] Davey KJ (2013). Antipsychotics and the gut microbiome: olanzapine-induced metabolic dysfunction is attenuated by antibiotic administration in the rat. Transl. Psychiatry.

[17] Lukić I (2019). Antidepressants affect gut microbiota and *Ruminococcus flavefaciens* is able to abolish their effects on depressive-like behavior. Transl. Psychiatry.

[18] Lyte M, Daniels KM, Schmitz-Esser S (2019). Fluoxetine-induced alteration of murine gut microbial community structure: evidence for a microbial endocrinology-based mechanism of action responsible for fluoxetine-induced side effects. PeerJ.

[19] Gimenez-Bastida JA, Martinez Carreras L, Moya-Pérez A, Laparra Llopis JM (2018). Pharmacological efficacy/toxicity of drugs: a comprehensive update about the dynamic interplay of microbes. J. Pharm. Sci..

[20] Qu Y (2017). Comparison of (R)-ketamine and lanicemine on depression-like phenotype and abnormal composition of gut microbiota in a social defeat stress model. Sci. Rep..

[21] Maier L (2018). Extensive impact of non-antibiotic drugs on human gut bacteria. Nature.

[22] Mandal A, Sinha C, Kumar Jena A, Ghosh S, Samanta A (2010). An investigation on in vitro and in vivo antimicrobial properties of the antidepressant: amitriptyline hydrochloride. Braz. J. Microbiol. Publ. Braz. Soc. Microbiol..

[23] Csiszar K, Molnar J (1992). Mechanism of action of tricyclic drugs on *Escherichia coli* and *Yersinia enterocolitica* plasmid maintenance and replication. Anticancer Res..

[24] Dean SN, van Hoek ML (2015). Screen of FDA-approved drug library identifies maprotiline, an antibiofilm and antivirulence compound with QseC sensor-kinase dependent activity in *Francisella novicida*. Virulence.

[25] Wilson TJ, Blackledge MS, Vigueira PA (2018). Resensitization of methicillin-resistant *Staphylococcus aureus* by amoxapine, an FDA-approved antidepressant. Heliyon.

[26] Bohnert JA, Szymaniak-Vits M, Schuster S, Kern WV (2011). Efflux inhibition by selective serotonin reuptake inhibitors in *Escherichia coli*. J. Antimicrob. Chemother..

[27] Ayaz M (2015). Citalopram and venlafaxine differentially augments antimicrobial properties of antibiotics. Acta Pol Pharm.

[28] Munoz-Bellido JL, Munoz-Criado S, Garcıa-Rodrıguez JA (2000). Antimicrobial activity of psychotropic drugs Selective serotonin reuptake inhibitors. Int. J. Antimicrob. Agents.

[29] Kalayci S, Demirci S, Sahin F (2015). Antimicrobial properties of various psychotropic drugs against broad range microorganisms. Curr. Psychopharmacol..

[30] Kromann S (2017). Treatment with high-dose antidepressants severely exacerbates the pathological outcome of experimental *Escherichia coli* infections in poultry. PLoS ONE.

[31] Ramsteijn AS, Jašarević E, Houwing DJ, Bale TL, Olivier JD (2020). Antidepressant treatment with fluoxetine during pregnancy and lactation modulates the gut microbiome and metabolome in a rat model relevant to depression. Gut Microbes.

[32] Andersson DI, Hughes D (2014). Microbiological effects of sublethal levels of antibiotics. Nat. Rev. Microbiol..

[33] Mazmanian SK, Kasper DL (2006). The love–hate relationship between bacterial polysaccharides and the host immune system. Nat. Rev. Immunol..

[34] O’Callaghan A, van Sinderen D (2016). Bifidobacteria and their role as members of the human gut microbiota. Front. Microbiol..

[35] Bahr SM (2015). Use of the second-generation antipsychotic, risperidone, and secondary weight gain are associated with an altered gut microbiota in children. Transl. Psychiatry.

[36] Flowers SA, Evans SJ, Ward KM, McInnis MG, Ellingrod VL (2017). Interaction between atypical antipsychotics and the gut microbiome in a bipolar disease cohort. Pharmacother. J. Hum. Pharmacol. Drug Ther..

[37] Plovier H (2017). A purified membrane protein from *Akkermansia muciniphila* or the pasteurized bacterium improves metabolism in obese and diabetic mice. Nat. Med..

[38] Liśkiewicz P (2019). Fecal microbiota analysis in patients going through a depressive episode during treatment in a psychiatric hospital setting. J. Clin. Med..

[39] Donaldson GP, Lee SM, Mazmanian SK (2016). Gut biogeography of the bacterial microbiota. Nat. Rev. Microbiol..

[40] Wishart DS (2018). DrugBank 5.0: a major update to the DrugBank database for 2018. Nucleic Acids Res..

[41] Schiller C (2005). Intestinal fluid volumes and transit of dosage forms as assessed by magnetic resonance imaging. Aliment. Pharmacol. Ther..

[42] Pritchard SE (2014). Fasting and postprandial volumes of the undisturbed colon: normal values and changes in diarrhea-predominant irritable bowel syndrome measured using serial MRI. Neurogastroenterol. Motil..

[43] Pratt LA, Brody DJ, Gu Q (2017). Antidepressant use among persons aged 12 and over: United States, 2011–2014. NCHS Data Brief.

[44] Vich Vila A (2020). Impact of commonly used drugs on the composition and metabolic function of the gut microbiota. Nat. Commun..

[45] Spanogiannopoulos P, Bess EN, Carmody RN, Turnbaugh PJ (2016). The microbial pharmacists within us: a metagenomic view of xenobiotic metabolism. Nat Rev Microbiol.

[46] Peppercorn MA, Goldman P (1972). The role of intestinal bacteria in the metabolism of salicylazosulfapyridine. J Pharmacol Exp Ther.

[47] Mason BL (2020). Reduced anti-inflammatory gut microbiota are associated with depression and anhedonia. J. Affect. Disord..

[48] Hammami R (2009). Antimicrobial properties of aqueous extracts from three medicinal plants growing wild in arid regions of Tunisia. Pharm. Biol..

[49] CLSI. *Methods for dilution antimicrobial susceptibility tests for bacteria that grow aerobically; approved standard*. M07-A11 (2018).

[50] CLSI. *Methods for Antimicrobial Susceptibility Testing of Anaerobic Bacteria*. M11-A9 (2018).

