# Supplementary material for: Unravelling the antimicrobial action of antidepressants on gut commensal microbes
Source: Sci Rep. 2020 Oct 21;10:17878. doi: 10.1038/s41598-020-74934-9 (PMC7578019; doi:10.1038/s41598-020-74934-9)
Supplement: Supplementary file 1 — Supplementary Information. [file 41598_2020_74934_MOESM1_ESM.pdf]

Supplementary file:

# Unravelling the antimicrobial action of antidepressants on gut commensal microbes

**Yasmina Ait Chait[1], Walid Mottawea[1,2], Thomas A. Tompkins[3], Riadh Hammami[1*]**

[1]N*u*Gut Research Platform, School of Nutrition Sciences, Faculty of Health Sciences, University of Ottawa, Ottawa, Ontario, K1N 6N5, Canada.

[2] Department of Microbiology and Immunology, Faculty of Pharmacy, Mansoura University, Mansoura, Egypt.

[3]Rosell Institute for Microbiome and Probiotics, Montreal, Quebec, H4P 2R2, Canada.

*Corresponding author: riadh.hammami@uottawa.ca

**Table S1.** Selected intestinal bacterial strains for the investigation of antibacterial activity.

| **Bacterial intestinal strains** | **Culture medium** |
|---|---|
| *Limosilactobacillus reuteri* ATCC 23272 | de Man Rogosa et Sharp (MRS) |
| *Lacticaseibacillus casei* ATCC 393 | MRS |
| *Lacticaseibacillus rhamnosus* ATCC 53103 | MRS |
| *Bifidobacterium animalis subsp. animalis* ATCC 25527 | MRS |
| *Enterococcus faecium* ATCC 35667 | MRS |
| *Pseudomonas aeruginosa* ATCC 27853 | Brain Heart Infusion (BHI) |
| *Escherichia coli* ATCC 25922 | BHI |
| *Akkermansia muciniphila* ATCC BAA-835 | BHI + 0.5% mucin |
| *Eubacterium rectale* ATCC 33656 | Fastidious Anaerobe Broth (FAB) + 0.5% yeast extract |
| *Faecalibacterium prausnitzii* ATCC 27768 | FAB + 0.5% yeast extract |
| *Bacteroides fragilis* ATCC 25285 | FAB + 0.5% yeast extract |
| *Clostridium leptum* ATCC 29065 | FAB + 0.5% yeast extract |

**Table S2.** Solvents used for psychotropics' stock solutions

| **Psychotropics** | **Solvent** |
|---|---|
| Phenelzine | Water |
| Venlafaxine | Dimethylsulfoxide |
| (S)-Citalopram | Methanol |
| Desipramine | Water |
| Bupropion | Methanol |
| Aripiprazole | Acetic acid |

**Table S3.** Inhibition zones diameters (mm) of some psychotropics against bacterial strains

| Psychotropic / Strain | Desipramine | | | Aripiprazole | | | Phenelzine | | | (S)-Citalopram | | |
|---|---|---|---|---|---|---|---|---|---|---|---|---|
| **Concentration (mg/ml)** | **10** | **5** | **2.5** | **10** | **5** | **2.5** | **10** | **5** | **2.5** | **10** | **5** | **2.5** |
| *L. rhamnosus* ATCC 53103 | 13 ± 0.22 | 10 ± 0.12 | NI | 24 ± 0.10 | 9 ± 0.11 | NI | 13 ± 0.12 | NI | NI | 9 ± 0.10 | NI | NI |
| *B. animalis* ATCC 25527 | 25 ± 0.12 | 18 ± 0.20 | NI | 19 ± 0.11 | 9 ± 0.11 | NI | 12 ± 0.22 | NI | NI | NI | NI | NI |
| *E. faecium* ATCC 35667 | 14 ± 0.21 | 11 ± 0.21 | 9 ± 0.10 | 17 ± 0.12 | 12 ± 0.21 | 9 ± 0.11 | 22 ± 0.21 | 17 ± 0.12 | 9 ± 0.14 | 14 ± 0.11 | 10 ± 0.12 | NI |
| *E. rectale* ATCC 33656 | 21 ± 0.13 | 16 ± 0.11 | 10 ± 0.12 | 18 ± 0.12 | 13 ± 0.11 | 11 ± 0.21 | 12 ± 0.11 | 14 ± 0.10 | 9 ± 0.12 | 11 ± 0.12 | NI | NI |
| *F. prausnitzii* ATCC 27768 | 22 ± 0.21 | 18 ± 0.11 | 15 ± 0.21 | 21 ± 0.10 | 17 ± 0.22 | 14 ± 0.11 | 20 ± 0.14 | 16 ± 0.11 | 13 ± 0.11 | 13 ± 0.11 | 9± 0.11 | NI |
| *B. fragilis* ATCC 25285 | 20 ± 0.22 | 17 ± 0.20 | 14 ± 0.11 | 19 ± 0.11 | 15 ± 0.12 | 12 ± 0.12 | 13 ± 0.21 | 9 ± 0.10 | NI | 12 ± 0.21 | 9 ± 0.13 | NI |
| *E. coli* ATCC 25922 | 21 ± 0.12 | 18 ± 0.12 | 16 ± 0.12 | 15 ± 0.11 | 12 ± 0.12 | 9 ± 0.11 | 19 ± 0.20 | 14 ± 0.22 | 10 ± 0.22 | 16 ± 0.12 | 11 ± 0.21 | 9 ± 0.10 |
| *C. leptum* ATCC 29065 | 35 ± 0.10 | 29 ± 0.11 | 22 ± 0.21 | 31 ± 0.12 | 22 ± 0.22 | 19 ± 0.21 | 17 ± 0.11 | 14 ± 0.21 | 9 ± 0.23 | 20 ± 0.11 | 15 ± 0.22 | 11 ± 0.11 |
| *A. muciniphila* ATCC BAA-835 | 32 ± 0.20 | 27 ± 0.12 | 20 ± 0.23 | 30 ± 0.13 | 22 ± 0.21 | 18 ± 0.22 | 21 ± 0.12 | 13 ± 0.12 | 10 ± 0.11 | 18 ± 0.13 | 14 ± 0.14 | 9 ± 0.13 |

NI: no inhibition detected

**Table S4.** Approximate concentrations of antidepressants reaching the human colon.

| Drugs | Preclinical/clinical (mg / day) (min – max) | % reaching the colon[1] | Concentrations reaching the colon (µg/mL) | | Tested concentrations (µg/mL) |
|---|---|---|---|---|---|
| | | | Colon volume 1* (min-max) | Colon volume 2** (min – max) | |
| Phenelzine | 45 – 75 | 21 | 727 - 1212 | 20-33 | 75-800 |
| Venlafaxine | 75 – 225 | 13 | 750 - 2250 | 20-61 | 75-800 |
| (S)-Citalopram | 20 – 60 | 10 | 154 - 462 | 16-188 | 75-800 |
| Desipramine | 25 – 300 | 30 | 577 - 6923 | 21-94 | 75-800 |
| Bupropion | 100 - 450 | 10 | 769 - 3462 | 11-34 | 75-800 |
| Aripiprazole | 10 – 30 | 55 | 423 - 1269 | 4-13 | 75-800 |

[1]source: https://www.drugbank.ca

*: Colon fluid volume = 18 mL, according to [41]

**: Colon volume = 480 mL, according to [42]

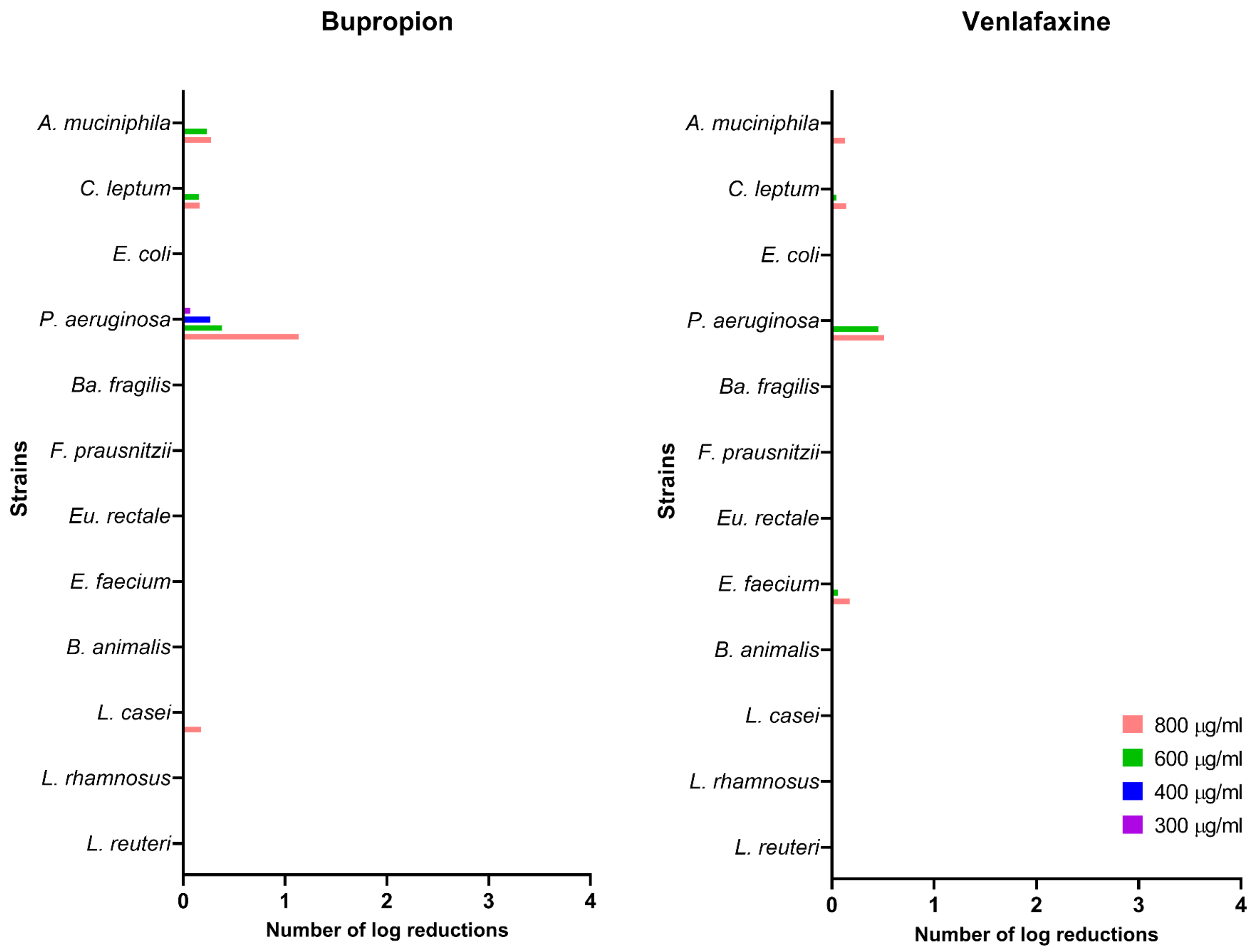


**Figure S1.** Logarithmic reductions of the growth of the commensal strains in the presence of bupropion and venlafaxine.

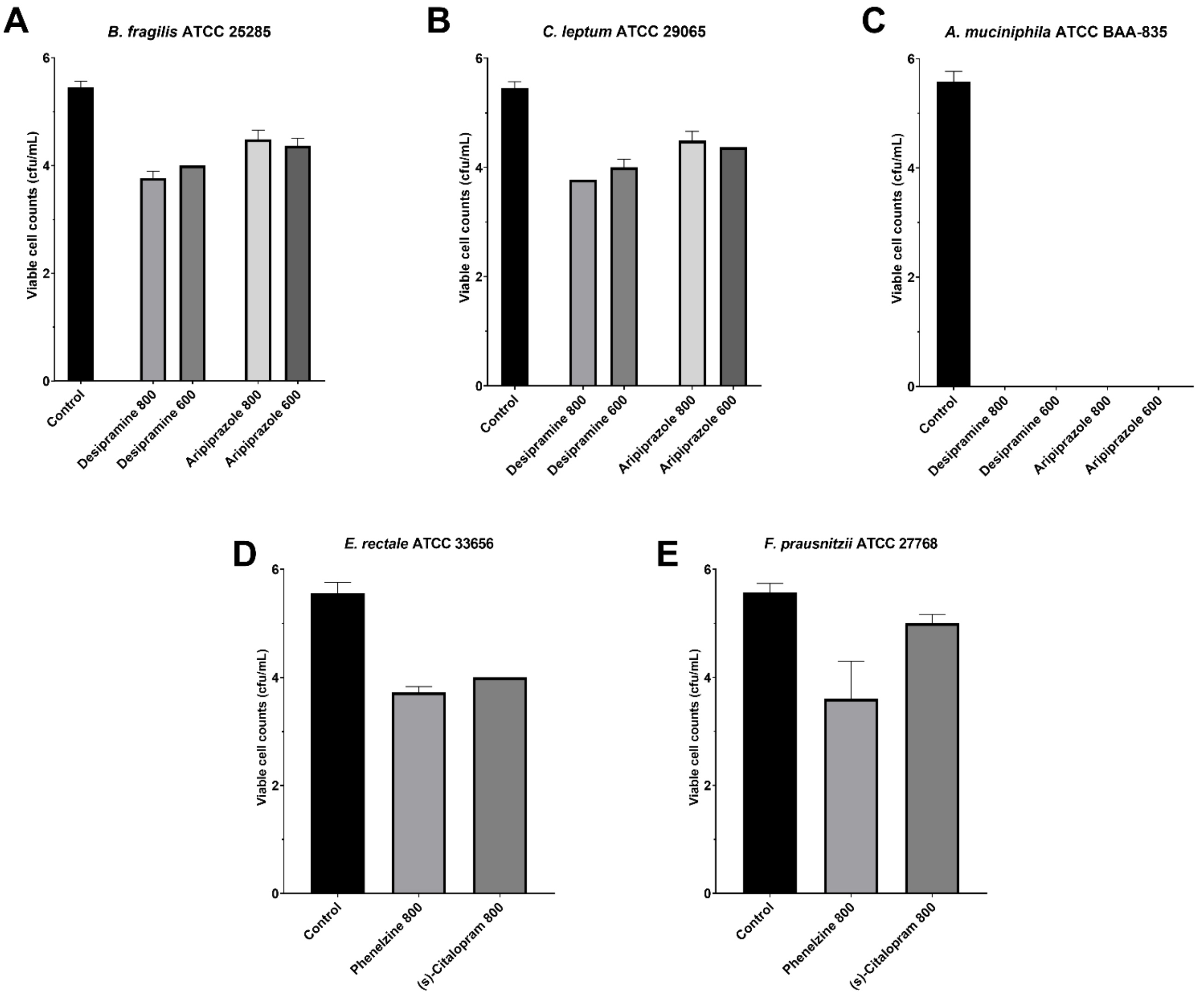


**Figure S2.** Bactericidal effect of desipramine, aripiprazole, phenelzine, and (s)-citalopram incubated for 16h at 600 and 800 µg/mL against tested bacterial strains. All treatments were statistically significant compared to control, which corresponds to initial inoculum cell counts (cfu/mL).

## REFERENCES


41. Schiller, C. *et al.* Intestinal fluid volumes and transit of dosage forms as assessed by magnetic resonance imaging. *Aliment. Pharmacol. Ther.* **22**, 971–979 (2005).
42. Pritchard, S. E. *et al.* Fasting and postprandial volumes of the undisturbed colon: normal values and changes in diarrhea-predominant irritable bowel syndrome measured using serial MRI. *Neurogastroenterol. Motil.* **26**, 124–130 (2014).
